# Development in Assay Methods for *in Vitro* Antimalarial Drug Efficacy Testing: A Systematic Review

**DOI:** 10.3389/fphar.2017.00754

**Published:** 2017-10-23

**Authors:** Shweta Sinha, Phulen Sarma, Rakesh Sehgal, Bikash Medhi

**Affiliations:** ^1^Department of Medical Parasitology, Postgraduate Institute of Medical Education and Research, Chandigarh, India; ^2^Department of Pharmacology, Postgraduate Institute of Medical Education and Research, Chandigarh, India

**Keywords:** malaria, *in vitro*, assay method, HTS, stem cells

## Abstract

The emergence and spread of drug resistance are the major challenges in malaria eradication mission. Besides various strategies laid down by World Health Organization, such as vector management, source reduction, early case detection, prompt treatment, and development of new diagnostics and vaccines, nevertheless the need for new and efficacious drugs against malaria has become a critical priority on the global malaria research agenda. At several screening stages, millions of compounds are screened (1,000–2,000,000 compounds per screening campaign), before pre-clinical trials to select optimum lead. Carrying out *in vitro* screening of antimalarials is very difficult as different assay methods are subject to numerous sources of variability across different laboratories around the globe. Despite this, *in vitro* screening is an essential part of antimalarial drug development as it enables to resource various confounding factors such as host immune response and drug–drug interaction. Therefore, in this article, we try to illustrate the basic necessity behind *in vitro* study and how new methods are developed and subsequently adopted for high-throughput antimalarial drug screening and its application in achieving the next level of *in vitro* screening based on the current approaches (such as stem cells).

## Introduction

Malaria is known for millennium for causing fatal consequences. Basically, it is caused by five different species of *Plasmodium* namely *falciparum, vivax, malariae, ovale*, and *knowlesi*. According to WHO there were 214 million cases of malaria across the world in the year 2015. Although the incidence and mortality decreased by 37 and 60%, respectively, globally between the years 2000 and 2015 (WHO, 2016) because of the continuous tremendous effort in the malaria eradication program and various other strategies, still more attention is required to obscure resistance phenomena of the diseases (Hyde, 2007; Sinha et al., 2014). Out of the various strategies to control over malaria cases, drug development organizations are also trying to develop a better, more efficacious and safe drug. Each year, thousands and thousands of moieties are screened for their antimalarial activity, but very few of them are capable of entering the market. Developing a whole new drug from a basic idea is so complex that it takes around 12–15 years and costs more than $1 billion till the launch of a new drug as a finished product in the market (Hughes et al., 2011). Recently, more emphasis is given to discovering new antimalarial drugs as a result of higher prevalence of resistance to most of the known antimalarial drugs in Southeast Asian countries (Noedl et al., 2008; Dondorp et al., 2009; Phyo et al., 2012) and to overcome this situation various academic as well as non-profit institutions are signing agreement with major pharmaceutical industry to develop some new antimalarial drugs. Moreover, there are few new antimalarial agents that are under clinical trial procedures and many of these agents are those which were resurrected from earlier antimalarial drug discovery programs (Gelb, 2007). Nowadays, the main challenge in drug discovery procedure is to streamline the whole method to reduce the manpower, total mechanistic energy and cost consumption, which begins with screening of thousands of compounds at *in vitro* level, which will reduce the burden at *in vivo* level and finally decrease the number of animals used for *in vivo* screening. Many variations in the *in vitro* antimalarial assay methods from the year 1968 to till date have arisen from a very basic concept called “macrotechnique” (Rieckmann et al., 1968). Subsequently, with advancement in knowledge regarding parasite biology, material science, and technology, there are a number of *in vitro* antimalarial assay methods that are used to screen antimalarials acting not only at the erythrocytic stage but also at the liver stage and the gametocyte stages. Most of these *in vitro* efficacy models assist us with direct knowledge toward any potential new drug/disease, serendipitous identification of new moieties in less time with minimum accountability and enable to determine antimalarial resistance patterns (Fidock et al., 2004). Also, screening of compounds *in vitro* in whole parasite assays is desirable as it helps in effective penetration of compound inside cellular membranes of the parasite to a measurable extent, which gives a strong basis for drug discovery by mimicking the *in vivo* situation (Hernandez et al., 2006; Hobbs and Duffy, 2011). However, because of the trend of adopting independent approaches by various research laboratories for developing their own assays, it often results in many variations relating to laboratory-practices, assays, and data-related variables. Therefore, it is recommended to screen new compounds applying multiple technologies to minimize or overcome these variations (Lucantoni et al., 2017). Therefore, in this article we try to illustrate the basic necessity behind *in vitro* study and how new methods are developed and subsequently adopted for high throughput antimalarial drug screening and its application in achieving the next level of *in vitro* screening based on the current approaches (such as stem cells).

## Materials and Methods

An electronic systematic literature search was accomplished to find all relevant studies in PubMed, EMBASE and Google Scholar starting from the year 1968 to till date. The search keywords and phrases included, *In vitro* models for antimalarial efficacy, antimalarial assay methods, antimalarial drug sensitive assay, *In vitro* efficacy models for *Plasmodium* and high throughput antimalarial drug screening. All *in vitro* methods that were mostly used for compound/drug screening/drug susceptibility testing were included in the study. However, studies involving *in vitro* methods for the clinical diagnosis such as rapid diagnostic techniques, Raman spectroscopy and HTS methods for other parasites were excluded. Unpublished data and thesis work were also excluded. For eliminating duplicity the title and abstract of all searched studies were examined twice and the reference lists of the selected articles were additionally reviewed for more relevant studies.

## Results

**FIGURE 1:**
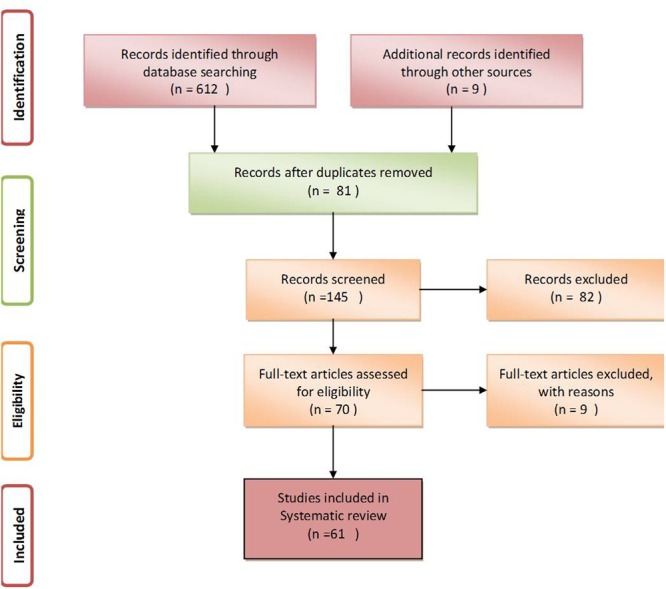
Schematic flow diagram to show selection criteria for systematic review.

After a thorough search of PubMed, EMBASE and Google Scholar, a total of 612 studies relevant to the assumed idea were retrieved. Among these, only 61 articles having appropriate content and those that fulfilled the inclusion and exclusion criteria were further selected for writing this review (**Figure 1**, Liberati et al., 2009).

## Discussion

**FIGURE 2:**
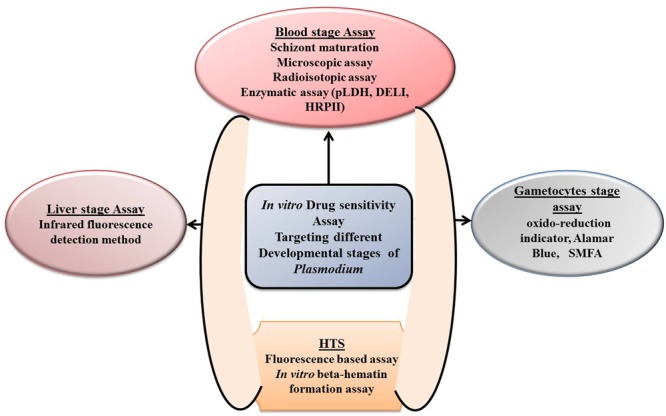
Depiction of different *in vitro* drug sensitivity assays targeting different development stages of *Plasmodium*.

Malaria parasite displays a complex life cycle which is intermediate between two hosts, a female Anopheles mosquito, which carries sporozoites in its salivary gland and later injects it into the human being while taking a blood meal. The next stage of malaria parasite begins with in the human being; it is divided into two phases that is, the pre-erythrocytic (exoerythrocytic) phase and the erythrocytic phase. It takes less than a minute for sporozoites to invade liver cells through blood circulation after being inoculated. Inside the liver cells, sporozoites transform into multi-nucleated schizonts. After that, these schizont releases thousands of merozoites (exoerythrocytic schizogony) into the peripheral circulation which is responsible for most of the clinical symptoms in the affected population. Understanding the parasite life cycle, parasite biology, and pathophysiology of the disease is the basic to know before any drug discovery effort. Drug efficacy test at *in vitro* level is the most preliminary step for screening any new compound libraries (**Figure 2**). Rieckmann et al. (1968) developed simple *in vitro* susceptibility tests called “macrotechnique” with an objective to find and follow the trend of evolution of chloroquine-resistant *Plasmodium falciparum* in several parts of the world. The method involves counting of schizonts in test vials with a comparison to drug-free controls and only because of its simplicity a standard test kit and a standard procedure, known as “WHO standard macrotest,” were established under the sponsorship of WHO. However, because of less reproducibility in the data, the assay was abandoned in the late 1980s (Rieckmann et al., 1968; Basco, 2007). Trager and Jensen (1976) illustrated a new *in vitro* method for continuous cultivation of *P. falciparum*, and with the introduction of this method, substantial modification in the principle of the previous practicing *in vitro* culture technique was undertaken (**Table 1**). Also, this modified technique seems to be pioneered in establishing the base of many *in vitro* drug susceptibility assays for screening thousands of drug moieties.

**Table 1:** Different *in vitro* drug sensitivity assays used in antimalarial drug screening.

*In vitro* efficacy models	Introductory year	Advantages	Disadvantages	Reliability/sensitivity	Reference
Macrotechnique	1968	• Simple, • Does not require sophisticated equipment • Reliable for field application	• Need for 10 ml of venous blood • Low success rate (<70%)	• A parasite count of between 1000 and 80, 000 asexual parasites per μl • Less reliable with poor sensitivity	Rieckmann et al., 1968
Microtechnique	1978	• Simple, • Small volume of blood • Does not require sophisticated equipment • Reliable for field application	• Requirement of trained personnel • Time consuming	• Moderate success rate • Moderate sensitivity	Rieckmann et al., 1978
Semiautomated microdilution technique or isotopic assays (radiolabeled hypoxanthine incorporation assay)	1979	• A rapid and quantitative measurement of antimalarial activity • Automatic reading • Reduces the chance of result variability	• Expensive • Time consuming • Multiple processing steps • Special handling and requirement of waste disposal system • Requirement for a relatively high (i.e., ≥0.1%) starting parasitemia • Inappropriate for field application • Requirement of instrument such as liquid scintillation counters and harvesting machines	• Reliability and sensitivity are moderate	Desjardins et al., 1979; Chulay et al., 1983.
Autometric flow cytometeric analysis	1990	• Fast • Automated • Accurate • Ability to differentiate between other parasite stages • Yields more information within one single analysis	• Expensive • Requirement of instrument	• Moderate reliability with high sensitivity	van Vianen et al., 1990; van Vianen et al., 1993
Isotopic assay (ethanolamine incorporation assay)	1992	• Fast • Reproducible • Automated with little data variability • Addition of hypoxanthine for additional parasite growth	• Expensive • Requirement of instrument • Special handling and requirement waste disposal system	• Reliability and sensitivity are moderate	Elabbadi et al., 1992
Lactate dehydrogenase (pLDH) assay	1993	• Fast • Reproducible • Automated with little data variability • No need of trained personnel	• Less applicable for field application • Need of initial parasitemia of 1–2%	• Reliable and very sensitive	Makler et al., 1993
Double-site enzyme-linked lactate dehydrogenase enzyme immunodetection (DELI) assay	2001	• Easier to perform • Faster to implement • No trained personnel needed • Reliable for field application • Cheaper than *in vitro* isotopic assays	• Expensive • Requirement of monoclonal antibody • Not reliable for field application	• Highly reliable and very sensitive to detect low parasitemia (0.005%)	Brasseur et al., 2001; Druilhe et al., 2001
Histidine-rich protein II (HRPII) drug susceptibility assay	2002	• Fast • Simple to establish • Highly reproducible	• Time consuming as it uses a longer culture time (72 h)	• Reliable and very sensitive	Noedl et al., 2002
		• Easy to perform • Useful to test slow acting drugs • Requires little technical equipment			
CyQUANT assay	2004	• Easier • More convenient to perform • Automated • Applicable for HTS • Requires fewer steps • Uses simpler equipment • Plate can be stored frozen for a long period before the assay	• Expensive	• Reliable and very sensitive	Sriwilaijaroen et al., 2004
Malaria SYBR Green I-based fluorescence (MSF) assay	2004	• Useful in resource-limited environment • Applicable for studying drug interaction in drug combinational studies in a research setting • Also applicable for clinical setting • Designed for HTS	• Expensive • Requirement of instruments	• Reliability and sensitivity are high	Smilkstein et al., 2004
Miniaturized pLDH-based growth inhibition assay (GIA assay)	2008	• Robust • Fast • Automated monitoring of *Plasmodium* growth in HTS.	• Expensive • Requirement of equipments	• Reliable and highly sensitive	Bergmann-Leitner et al., 2008
Non-radioactive DAPI-based high-throughput *in vitro* assay	2007, 2010	• Robust • Compatibility of the fluorescent dye, DAPI for monitoring *Plasmodium* growth in HTS	• Expensive • Instrument requirement	• Reliable and highly sensitive	Baniecki et al., 2007; Ndiaye et al., 2010
Transgenic parasites expressing reporter genes	2007, 2012	• Simple • No significant differences between synchronized and unsynchronized parasites • Performed within 12 h for fast-acting drugs	• Expensive • Requirement of transfection technology	• Reliable and sensitive as the standard radioisotope incorporation method	Cui et al., 2007; Khan et al., 2012
Flow cytometric hemozoin detection assay	2013	• Take less time	• Expensive	• Reliable and sensitive	Rebelo et al., 2013
Luciferase-based high-throughput screening (HTS) assay	2013	• Robust • Better signal-to-noise ratios • Wide dynamic range	• FACS is required for analysis, rather than simple fluorescence microplate readers • Analysis of GFP fluorescence needs to be performed on live parasites and ring-form stage parasites that contain a single-copy gfp gene, which is difficult for distinguishing from uninfected GFP-fluorescence cells.	• Moderate reliability and superior sensitivity	Lucantoni et al., 2013

## Schizont Counting Based On Simple Microscopy

After a series of modifications in 48 h variant test, Rieckmann et al. (1978) developed “Microtest”. Briefly, after preparation of thick blood smears from each well, the total number of schizonts in each well was counted against 500 leukocytes. Finally, *in vitro* activity was demonstrated as the percentage of the total counted schizonts at each drug concentration, with regard to the total counted schizonts in drug-free controls. Fingerprick capillary blood samples are sufficient to do this test. After that, the technique was later adopted to design a field-relevant microtest, under the sponsorship of WHO by Wernsdorfer and Kouznetsov (1980), Wernsdorfer and Payne (1988). Basically, macrotest and microtest are the two earlier WHO assay systems that are designed for using as laboratory tools to assist in surveillance and description regarding the epidemiology of drug-resistant malaria as part of the global monitoring program. These assay systems are used to follow the evolution pattern of drug resistance parasite and can also be used for determining the baseline levels of malaria drug sensitivity. On the basis of the above two protocols, Mark I, Mark II, and Mark III were developed between 1981 and 2000 which is known by “WHO standard *in vitro* Microtest kit” (Basco, 2007).

## Assay Based On Radioisotopes

During malaria parasite culture, platelets and uninfected erythrocytes in the culture plate do not synthesize their RNA, DNA, proteins or any of their membranes. Also, leukocytes are unable to multiply and hence disintegrate within a certain period of time. So, among all these, only malaria parasites have actively dividing cells. Hence, the addition of radioactive substances into the culture media enables the parasites to incorporate radioactive precursors themselves, which seems to be a sensitive indirect measurement assay for the parasite metabolic activity. Another beneficial thing is the reliability of *Plasmodium* sp. to use exogenous purines as they are unable to synthesize purines *de novo* (Sherman, 1977). Among radioisotopes, [^3^H] hypoxanthine is the most preferred radioisotope for antimalarial *in vitro* drug sensitivity assays as it is also the main purine base needed by *P. falciparum*. During this assay, under normal conditions, incorporation of [^3^H] hypoxanthine is directly related to the count of *P. falciparum*-infected erythrocytes and the initial parasitemia required for this assay is between 0.1 and 1.0% at 1.5% hematocrit during 42 h of incubation (Chulay et al., 1983; Geary et al., 1983). Radiolabeled precursors of phospholipids, such as the sources of phospholipid polar head groups, [^3^H] ethanolamine were incorporated by Elabbadi et al. (1992); [^3^H] ethanolamine is another radioisotope used for similar assay. However, incorporation was observed more with [^3^H] ethanolamine into infected erythrocytes as compared with [^3^H] hypoxanthine. During drug sensitivity assay both [^3^H] ethanolamine and [^3^H] hypoxanthine incorporation increases linearly at starting parasitemia in between 0.1 and 1% with haematocrit levels of 0.1–3%. Both these radioisotope precursors exhibit distinct metabolic activities and the findings suggest that, [^3^H] ethanolamine can be a better substitute for [^3^H] hypoxanthine to assess parasite viability. Also, similar responses under *in vitro* conditions with *P. falciparum* reference clone were obtained when [^3^H] ethanolamine radioisotope assay was compared with SYBR Green I-based fluoroassay (Smilkstein et al., 2004). The first radioisotope, semi-automated assay was that of Desjardins et al. (1979). The method was primarily adopted for drug screening in the United States Army Antimalarial Drug Development Program at the Walter Reed Army Institute of Research (Washington, DC, United States) and presently considered as the “gold standard” for various *in vitro* drug sensitivity assays. The assay involves the use of liquid scintillation counter for quantification of incorporated [^3^H] hypoxanthine. This assay method is now widely used in well-equipped laboratories and has also been adapted for studying epidemiology on fresh clinical isolates (Basco, 2007). Also, this assay is supposed to be a reference method in most of the advanced countries for assaying drug sensitivity; however, the same is not a reference method in most of the malaria-endemic countries (Basco, 2007). Stringent regulations regarding handling and disposal of radioactive material since the late 1970s and requirement of approximately 0.5% of parasitemia along with the compulsion to use highly expensive instruments, such as liquid scintillation counters, limit the test application for applying under field conditions (Noedl et al., 2003a). All these situations necessitate the use of non-radioactive methods as a standard method in near future.

## Enzyme-Based Assay

Parasite lactate dehydrogenase is an important terminal enzyme of the glycolytic pathway in *Plasmodium* parasite and thus plays a vital role in anaerobic carbohydrate metabolism (Makler et al., 1993). *Plasmodium* chiefly depends on anaerobic glycolysis, and for this they need regeneration of NAD to get a continuous flux of glucose through this pathway (Sherman, 1998). As an enzyme structure of pLDH is morphologically different from host LDH, so its production and accumulation are used as indices for checking parasite viability (Sherman, 1961; Makler et al., 1993; Brown et al., 2004). Hence, a drug-sensitivity assay, which displays inhibitory profiles of parasite metabolic activity through estimation of enzyme pLDH was developed by Makler and Hinrichs (1993). The assay is rooted on monitoring the ability of LDH enzyme to quickly use a coenzyme, APAD which is a NAD analog, in reaction, that converts lactate into pyruvate. However, LDH of the host erythrocytes carries out the same reaction at a very slow pace in the presence of APAD. In the assay, development of reduced APAD (APADH) is measured, which interprets a direct correlation between parasitemia level and pLDH activity (Makler and Hinrichs, 1993; Makler et al., 1993; Basco et al., 1995). Because of limitations such as the requirement of high parasite densities of 1–2% and insensitivity for field applications, a DELI assay was developed, which was based on monoclonal antibodies specific for pLDH and this assay was found to be applicable for both diagnostic and drug-sensitivity testing. The technique is highly sensitive as it can detect the parasite at very low level of parasitemia (<0.005%). However, because of limited availability of monoclonal antibodies specific to the pLDH, it has limited its application (Nogueira, 2010). The other, assay is based on water-soluble, histidine and alanine rich proteins, that is, HRP II which is mostly localized in various cellular compartments of parasite including cytoplasm (Howard et al., 1986). This assay is at least 10 times more sensitive than the other isotopic assays with minor technical requirements. The method involves measurement of HRP II levels, which is directly associated with parasite density and its growth (Desakorn et al., 1997; Noedl et al., 2002). Moreover, HRP II assay takes longer time (72 h) in culturing rather than other assays (48 h), which enables it an advantageous assay for testing of slow-acting drugs without incorporating any changes to the existing protocol (Noedl et al., 2002).

## High Throughput Screening (HTS)

Because of various limitations, such as the requirement of microscopist, data variation because of manual counting in the schizont maturation assay, use and disposal of radioactive substances in hypoxanthine assay, high cost, with several processing steps, along with less compatibility for HTS purpose, there was the development of assay in early 1990s based on flow cytometry analysis. The basic principle behind flow cytometry is that it takes advantage that human erythrocytes lack DNA so it stains and detects only parasite DNA. Briefly, the technology involves, incubation of parasites for a specified period with the test compounds, followed by fixation and then staining in which the whole parasitized cells can be stained with dye, hydroethidine or the nucleus of the parasite can be stained with DAPI, a fluorescent dye. Thereafter, flow cytometry can be used for counting treated and control cultures. Apart from this, flow cytometry also enables to differentiate between different stages of parasites inside erythrocyte with the help of gating. All these features make it a relatively simple assay, which gives high throughput and becomes the reason for replacement of older techniques, but the most important concern in using it, is its cost limitation. van Vianen et al. (1990), reported a fully automated analysis of drug tests by flow cytometry and they previously used flow cytometry for the screening of a newly developed drug. The flow cytometric method also determines schizont maturation but allows the exact number of nuclei per parasite to be determined, giving a more accurate account of the development in culture. Additionally, the parasitemia is counted in each well, so that a correction can be made for reinvasion. A variety of different experiments, designed to study drug sensitivity, invasion-blocking, optimization of culture conditions, and the comparative relative fitness of parasite populations, can be analyzed without any alterations of the culture procedures. Flow cytometry is more accurate in counting parasitemia, for instance, and can differentiate between developmental stages, which improve the reading of tests compared with conventional techniques. This can result in an increase in the number of successful tests, but it can also lead to the simplification of culture procedures. An additional improvement is the automation of the analysis and processing of the data. All data are processed in a standardized way, maintaining objectivity, and the data remain available for re-examination. Data from large numbers of tests can be combined. The results are directly available in tables and graphs, which are especially important in large studies.

## Fluorescence-Based Assay

Furthermore, the need for highly efficient throughput as well as the demand for non-radioactive assays resulted in the development of new fluorescence-based techniques which involves fast automatic quantification of parasite growth after staining parasites with fluorescent DNA binding dyes such as ethidium bromide (Waki et al., 1986). Binding of ethidium bromide to DNA causes the enhancement of its fluorescence intensity which is proportional to the amount of parasite DNA. This method was a replacement method to other radioactive assays in the late 1980s and was also applicable for screening antimalarials. Later a number of fluorescence dyes were used for HTS of drugs. Baniecki et al. (2007), revealed the compatibility and robustness for observing *Plasmodium* growth in HTS using DAPI, in a 384-well microtiter plate. Apart from this, few other DNA intercalating dyes, such as SYBR Green I, YOYO-1, and PicoGreen, have been recently described for measuring *in vitro Plasmodium* growth inhibition (Bennett et al., 2004; Smilkstein et al., 2004; Kosaisavee et al., 2006; Quashie et al., 2006; Weisman et al., 2006; Baniecki et al., 2007). As mature erythrocytes do not have RNA and DNA, the dye specifically binds to parasite DNA at the erythrocytic stage of *P. falciparum* and is significantly highly sensitive to well-defined spectral peaks, and is also less mutagenic when compared with ethidium bromide (Bennett et al., 2004). A study carried out by Smilkstein et al. (2004), demonstrated that the values of IC_50_ were the same with SYBR Green I as those obtained with the radioisotope, [^3^H] ethanolamine incorporation.

Recent advances in implication of transfection technology on malaria parasite enable the initiation of the transgenic type of parasite lines that express different reporters (Crabb, 2002), and it is found to be more sensitive like other standard radioactive incorporation assay with nearly zero background luminescence which does not require empty wells or uninfected erythrocytes as negative controls (Sanchez et al., 2007). Cui et al. (2008), generated stably expressing *P. falciparum* lines with a reporter firefly luciferase, which was optimized for 96-well microtiter plate format and serve as non-radiolabel-free, convenient system (Cui et al., 2007; Khan et al., 2012). Besides, the assay is simplified as it can use both unsynchronized and synchronized parasites with no significant differences, which suggest less time consumption and manpower. Moreover, most of the antimalarial assays take around 48–72 h to yield the results, however, luciferase assay takes only 12 h for interpreting the results and hence reliable for fast-acting drugs which suggest that expression of the reporter is altered before any parasite replicates. Although the assay is not fabricated for antimalarial resistance monitoring purposes, its simplicity indicates better feasibility of this luminescent assay for antimalarial HTS. Also, HTS requires at least 384-well microtiter culture plate and this assay can be performed in 384-well or even higher format as Z′ scores of >0.77 supports for 384-well assay (Cui et al., 2008). The CyQUANT assay demonstrated by Sriwilaijaroen et al. (2004), is another fluorescence-based assay that uses CyQUANT GR cyanine dye that displays strong fluorescence, which can easily be detected at the excitation of 485 nm and visualized at 530 nm with a flow cytometer. The assay was found to be optimum for *in vitro* antimalarial assays and the GR dye shows optimum sensitivity and widest linearity range in comparison to other cyanine dyes (Sriwilaijaroen et al., 2004).

## *In Vitro* Beta-Hematin Formation Assay

Hemozoin is a non-toxic metabolite synthesized by the malaria parasite during heme metabolism that elucidates one of the distinct features of *Plasmodium*. Inhibition of heme metabolism leads to the acquisition of toxic heme that ultimately kills *Plasmodium* as a result of membrane lysis and hindrance in other metabolic functions in *Plasmodium* (Francis et al., 1997; Pandey et al., 1999; Stojiljkovic et al., 2001).

Briefly, for estimation of beta-hematin as a part of the antimalarial screening, the procedure involves beta-hematin formation which is initiated after addition of a catalytic factor in appropriate amount or nucleation. Thereafter, test compounds are added to the reaction mixtures followed by incubation for 12–24 h and finally the resulting beta-hematin is quantitated by different methods. (Tekwani and Walker, 2005). Most protocols involve differentiation of beta-hematin pellet through filtration or centrifugation followed by sequential washing of pellet with either water or solution of Tris-HCL/SDS and alkaline bicarbonate solution or DMSO. Besides, various experimental approaches are mentioned about beta-hematin estimation *in vitro* and use of this for evaluating antimalarial screening. Quantification of beta-hematin is the major step in this assay that can be done by a number of techniques such as spectrophotometric (Basilico et al., 1998; Pandey et al., 1999; Baelmans et al., 2000; Parapini et al., 2000; Tripathi et al., 2001, 2004; Deharo et al., 2002) radioisotopic (Kurosawa et al., 2000), fluorometric (Sullivan and Meshnick, 1996; Rebelo et al., 2013), and HPLC–based (Berger et al., 1995). Monomeric heme and hemozoin/beta-hematin may also be differentiated by FT-IR spectroscopy (Slater et al., 1991).

A rapid screening assay for antimalarial drugs has been developed from *Plasmodium* to determine deoxyhypusine and hypusine formed with the purified enzymes DHS and DOHH (Kaiser et al., 2012).

## *In Vitro* Assays Targeting Liver Stages

Gego et al. (2006) developed a new approach based on infrared fluorescence detection to automatically and rapidly quantify *Plasmodium* liver schizonts *in vitro*. Briefly, the technique involves plating of HepG2 or primary hepatocytes on plastic plates for about 24 h before inoculation of *Plasmodium* sporozoites, which follows short centrifugation, washing, and incubation of cells for 48 h for 5 days before quantification. Quantification was done through the Odyssey infrared imaging system combined with a colony counter, and this approach was validated against three strains of *P. berghei* (ANKA strain), *P. yoelii* (265BY strain), and *P. falciparum* (NF54 strain) (Gego et al., 2006).

## *In Vitro* Assay for Gametocytes

Gametocytes of *P. falciparum* undergoes five morphologically separate indistinguishable stages within erythrocytes over a period of between 8 and 12 days (Baker, 2010) and then they retain in peripheral blood for few weeks (Bousema et al., 2010). Gametocyte stage represents “druggable” transmission-related stage of *Plasmodium* and therefore there are so many *in vitro* methods that have been studied and published for assessing the gametocidal activity of compounds (Lucantoni and Avery, 2012). Most of these assays are based on metabolic activity measurement (Peatey et al., 2011), which is directly correlated with the intensity of fluorescence dye emitted from transgenic parasite lines that express a kind of reporter gene (Buchholz et al., 2011), alamar Blue, an oxidoreduction indicator (Tanaka and Williamson, 2011), and bioluminescence-mediated detection of ATP (Lelièvre et al., 2012) and also, pLDH activity is studied recently (Roncales et al., 2012). Later, Tanaka et al. (2013) modified and validated alamar Blue indicator assay suited for HTS and to assess the viability, gametocytes are also added to this concept. All the above methods mainly focused on gametocytogenesis at later steps of development, as immature stages are often hypothesized for their sensitivity to most of the antimalarial drugs and, therefore, are of limited concern. Till now, there is no such study that produces an extensive assessment on the level of sensitivity at early-stage gametocytes with respect to most of the antimalarials, although there are few studies which are limited to few established drugs (Smalley, 1977; Chutmongkonkul et al., 1992; Fleck et al., 1996; Chavalitshewinkoon-Petmitr et al., 2000; Adjalley et al., 2011; Buchholz et al., 2011). Besides, previous and some recent reports illustrate the differences that exist with respect to the development of a different chemosensitivity pattern of immature developing gametocytes as well as asexual stages to several antimalarial compounds such as chloroquine (Chutmongkonkul et al., 1992), pyrimethamine (Chutmongkonkul et al., 1992) atovaquone (Smalley, 1977; Fleck et al., 1996; Adjalley et al., 2011), and methylene blue (Buchholz et al., 2011). D’Alessandro et al. (2013) reported the fabrication of novel, quick and cheapest assay for screening gametocidal compounds by measuring the activity of pLDH of gametocytes in 96-well plates. The assay validation was carried out using reference anti-gametocyte drugs, and the result was interpreted by comparing the readout measures of mosquito infectivity using the standard membrane feeding assay (SMFA), which is the current gold standard transmission blocking assay (van der Kolk et al., 2005; Stone et al., 2014). Recently, an HTS assay applying luciferase approach has been performed in assessing compound activity on early (stages I–III) *P. falciparum*. The authors demonstrated the application of this assay with reference antimalarials and also reported few new leads from the MMV Malaria Box (Lucantoni et al., 2013). Lucantoni et al. (2016) extended their previous application to provide a prompt, straightforward, and cost-effective HTS assay, which is capable of assessing compound activity against late-stage gametocytes (stages IV and V), with the additional benefit of profiling activity throughout the process of gametocyte development and maturation. The assay is also suitable for evaluating compound activity at an incubation period of up to 72 h, which illustrates its excellent quality and reproducibility, having average *Z*′-values of 0.85 ± 0.01 (Lucantoni et al., 2016).

At present, new drugs that hinder both or either hepatocyte or gametocyte development, are needed to block parasite transmission. Thus, screening methods that are suitable for exploring new gametocidal compounds are needed for securing further transmission of malaria mostly in the endemic regions. However, a major drawback in the development of these assays is the unavailability of standardized protocols for gametocyte cultivation. However, there are few reference compounds against gametocyte development that mostly used for validation of new assays.

## Next-Generation Advances Assay

**FIGURE 3:**
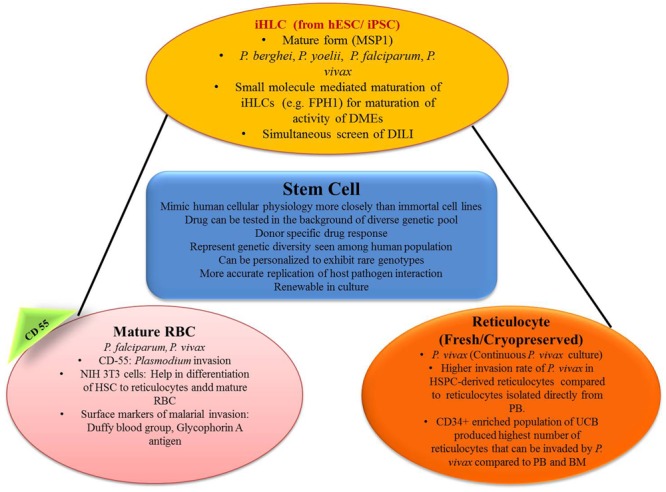
Role of stem cell in generating *in vitro* efficacy models for malaria. iHLC, induced human hepatocyte-like cells; hESC, human embryonic stem cells; MSP1, merozoite surface protein 1; FPH1, functional proliferation of primary hepatocytes 1; DMEs, drug metabolizing enzymes; DILI, drug-induced liver injury; CD55, clusters of differentiation; NIH 3T3, mouse embryonic fibroblast cell line; HSC, hematopoietic stem cells; HSPC, hematopoietic stem/progenitor cell; PB, peripheral blood; UCB, umbilical cord blood; BM, bone marrow.

In recent years, breakthroughs in the field of stem-cell research provide an additional opportunity for studying new prospectives in parasite biology, especially those concerned with the stages of the parasite cell cycle that seem to be challenging so far or even impossible task may seek through this new advances at *in vitro* level (**Figure 3**).

## Hepatic Stage Malaria: Role of Induced Pluripotent Cells

For the modeling of the hepatic stage of malaria, hepatocyte cell lines that is, HepG2, HCO4, are the commonly used ones, and the most commonly used malaria species are *Plasmodium* sporozoites *P. yoelii, P. berghei, P. falciparum*, and *P. vivax* (March et al., 2013). But metabolism in immortal cell lines is a little bit different than *in vivo* ones and thus they may not mimic *in vivo* conditions properly (Noulin, 2016). Again primary hepatocytes are the natural hosts of malaria (March et al., 2013). Primary rodent hepatocyte culture came into the scenario to solve this problem and *P. vivax* was successfully cultured in this model (Mazier et al., 1984). The use of human hepatocyte for the culture of malaria parasite was established by Dembélé et al. (2014). They successfully cultured exo-erythrocytic (EE) stages of *P. falciparum* and *P. cynomolgi in vitro* and were also able to develop hypnozoite form. This protocol may be very useful for the search of drugs that kill hypnozoites and also to study hypnozoite biology (Dembélé et al., 2014). But the use of primary hepatocyte brings another problem; that is, it needs continuous availability of fresh cells. The procedure for cryopreservation was established by March et al. (2013), and the use of this protocol permitted reuse of cryopreserved primary hepatocytes and these reused cultures showed evidence of *Plasmodium* invasion (March et al., 2013).

However, the primary hepatocytes are derived from a small pool of donors. So this small number of sample pool may not be representative of the genetic diversity seen in human populations (Noulin, 2016). Hepatocytes derived from stem cells, overcome many of these limitations. Stem-cell-derived hepatocytes may represent more diverse genotypes; renewable and personalization can be done with the expression of rare genotypes (Ng et al., 2015).

Induced pluripotent stem cells (iPSCs) can be differentiated *in vitro* to produce iHLCs with many intermediate stages (pluripotent, definitive endoderm, specified hepatic, immature hepatocyte, mature hepatocyte) using the specific cell culture protocol as mentioned by Si-Tayeb et al. (2010). These stem cells can be collected from a huge pool of donors and this can give us a bank of iPSCs with huge genetic diversity (Ng et al., 2015). Ng et al. (2015) also tested whether iPSC-derived iHLCs can be used as a model for the hepatic stage of malaria or not. In their experiment, iHLCs, demonstrated typical hepatocyte morphology with a polygonal shape and expressed prototypical hepatocyte markers such as human albumin, α1-anti-trypsin, and α-fetoprotein. iHLCs were positive for both CD81 and SRB1, which are host entry factors for liver-stage malaria, and they were appropriately localized to the cell surface. In their experiment they found that the liver stage of *Plasmodium* can be effectively modeled using iPSC derived iHLCs. But it was found that iHLCs infected with *Plasmodium* were sensitive to atovaquone, but not to primaquine. Atovaquone is active against *Plasmodium* in the parent form, but primaquine requires bioactivation by the action of hepatic enzymes. In the next step of their experiment, they used small molecule (FPH1) to upregulate adult human drug metabolizing enzyme. In FPH1 treated, *Plasmodium* infected iHLCs, primaquine was effective against both *P. yoelii* and *P. falciparum* EEFs, (Ng et al., 2015). Therefore, this model can be an important part of antimalarial drug discovery with specifically targeting at the liver stage.

## Erythrocytic Phase

Giarratana et al. (2011) demonstrated a novel technique of generating erythrocytes *in vitro* from CD34+ HSCs. These erythrocytes were similar in terms of deformability, enzyme content, the capacity of their hemoglobin to fix/release oxygen, and expression of blood group antigens to normal endogenous erythrocytes. In a phase 1 study of the same, it was successfully translated into humans with a similar half-life (Giarratana et al., 2011). Fernandez-Becerra et al. (2013) showed that those erythrocytes which are acquired from either peripheral blood or bone marrow CD34+ human HSCs (hHSCs) are mostly permissive for *Plasmodium* infection. In their experiment, on day 14 growing erythrocytes showed expression of glycophorin A along with Duffy blood group antigen, which is considered as a surface marker for the invasion of *Plasmodium*. After that invasion assays were performed using 3D7 *P. falciparum* parasite strain and *P. vivax* isolates and it was demonstrated that erythrocytes and reticulocytes both differentiated from CD34+ hHSCs and were acquiescent for the invasion of both *Plasmodium* species (Fernandez-Becerra et al., 2013).

*Plasmodium falciparum* can invade erythrocytes of all stages, but *P. vivax* preferably invades reticulocytes (Golenda et al., 1997). But reticulocytes are only 0.5–1% of the total erythrocytes in the bloodstream and again their lifespan before maturation is only 24 h. Earlier methods used to concentrate reticulocytes were centrifugation (Golenda et al., 1997; Borlon et al., 2012), and use of lysis buffer (Grimberg et al., 2012). Recent studies clearly demonstrated the preference of *P. vivax* toward reticulocytes with high CD71 positive cells (maturation of reticulocytes is associated with selective removal of membrane proteins, e.g., transferrin receptor, CD71) (Martín-Jaular et al., 2013). This shows the possibility of HSC-derived reticulocytes in the drug discovery process against *P. vivax*. Panichakul et al. (2007) developed an *in vitro* method in which human cord HSCs were differentiated artificially in the presence of erythropoietin to produce susceptible erythrocyte precursors. Duffy positive reticulocytes appeared after 10 days and the maximum numbers were found in 14–16 days of culture. *P. vivax* was co-cultured on day 10 onward and parasitic growth was detected in growing erythrocytes. These cultures were maintained for >2 weeks to 85 days by supplying erythrocytes. Peak reticulocyte count (0.5%) was observed on day 14 and parasitemia was very less between 0.0001 and 0.0013% (Panichakul et al., 2007). Noulin et al. (2012) developed a method in which reticulocyte first appeared at day 12 of differentiation and the peak reticulocyte count was observed on day 14 (5% on day 13 and 18% on day 14). The reticulocyte count then decreased suddenly to 10% on day 16 which almost disappeared on day 19 (Noulin et al., 2012). Another problem was fresh reticulocytes were to be used along with fresh *vivax* isolates. Noulin et al. (2012) developed a modified technique of cryopreservation of HSC derived reticulocytes which can later be used for invasive tests which is an important step toward continuous *P. vivax* culture (Noulin et al., 2012).

## Conclusion

At the compound screening stage, many thousands of compounds are screened (1,000–2,000,000 compounds per screening campaign) using recent HTS techniques. Now, after identification of hits, which has an average of approximately 1.0%, they are supposed to be ranked which is based on several criteria (such as the way of synthesis, potency, toxicity; contradiction and other limitations and novelty to use) for finding out the most optimum leads. Most of the compounds are validated and tested in low throughput screening methods which include radical cure and transmission assays. However, both these are very expensive along with time-consumption and thus, they are mostly applied to the selective size of compounds. After leads selection, optimization for optimum characteristics such as maximum efficiency along with better bioavailability and less toxicity is taken into consideration. Finally, modified leads undergo further evaluation, and later those with maximum favorable risk-benefit ratio are forwarded to the next step of development; that is, preclinical evaluation and in case the lead is found successful, and then it may be carried into further clinical development phases.

Out of several *in vitro*/*ex vivo* susceptibility assays, the most demanding antimalarial assays includes, HRP-II ELISA, radioisotopic incorporation, and presently the SYBR Green I method and all are subjected to lots of data variabilities. The reason for these variabilities and confounding interpretations is the differences in protocol of assay methods, different laboratory conditions, use of distinct parasite strain for susceptibility phenotypes, and other differences arising because of individual technical performance (Noedl et al., 2003b; Bacon et al., 2007; Akala et al., 2011). Apart from the above conditions, drug properties, such as its solubility, pH difference, and mechanism of action display, method-dependent positive or negative effects on data analysis along with result interpretation (Wein et al., 2010). Also, it is challenging to extrapolate the results of *in vitro* testing to *in vivo* that is, intact systems biology (Rothman, 2002; De Clercq, 2005). Hence, more careful observation is required regarding to proper lead selection and optimization and *in vitro* toxicity profile. Again, in a drug requiring metabolic activation, *in vitro* evaluation techniques may be little difficult, although primary hepatocyte culture and other newer techniques such as the application of stem cells give insight on how to solve this problem (Li and Kedderis, 1997; Gómez-Lechón et al., 2003). Mathematical models and newer techniques such as “human on chip” may be helpful for better *in vitro–in vivo* correlation (Sung et al., 2010; Quignot and Bois, 2013). Apart from this, PB-PK models are important components to these extrapolations (Yoon et al., 2012).

However, *in vitro* tissue and organ sensitivity may be completely different to that what observed in cultured cells *in vitro* and thus *in vitro* cellular PK-PD profile may be different from *in vivo* cellular PK-PD profile. Therefore, PB-PK models are important components to these extrapolations (Yoon et al., 2012).

Irrespective of these deficiencies/disadvantages, *in vitro* techniques are cornerstones of the new drug discovery process. They provide us direct knowledge in relation to a potential new drug/disease setting; multiple compounds with different modes of action can be tested and, depending on the throughput of the assay, over a full concentration–effect range; it allows for some serendipity, as well as hypothesis-free, analysis of compounds which provides strong data-driven possibilities for subsequent assessment in highly complex phenotypic or *in vivo* systems. Some of the advantages that are offered by the *in vitro* efficacy models include the following:

• Simple
• Even human cells can be used for evaluation, so we can gather human data
• Convenience: large number of compounds can be evaluated at the same time
• Automation: as in case of HTS
• Precise and efficient
• Rapid
• Synergism or antagonism with drug combinations can be studied
• Better assessment of the intrinsic activity of a drug.

## Author Contributions

BM, RS, PS, and SS designed the layout of review. SS and PS collected the data. BM and RS analyzed the data. SS, PS, RS, and BM prepared the article.

## Conflict of Interest Statement

The authors declare that the research was conducted in the absence of any commercial or financial relationships that could be construed as a potential conflict of interest.

## Abbreviations

APAD: 3-acetylpyridine adenine dinucleotide
CD34+: cluster of Differentiation 34
CD71: cluster of Differentiation 71
CD81: cluster of Differentiation 81
DAPI, 4: 6-diamidino-2-phenylindole
DELI: double-site enzyme-Linked LDH immunodetection
DHS: deoxyhypusine synthase
DMSO: dimethyl sulfoxide
DOHH: deoxyhypusine hydroxylase
EEFs: exoerythrocytic forms
FPH1: functional proliferation of primary hepatocytes 1
FT-IR: Fourier transform infrared
hLDH: human lactate dehydrogenase
HPLC: high-performance liquid chromatography
HRP-II: histidine-rich protein II
HRP-II ELISA: histidine-rich protein II enzyme-linked immunosorbent assay
HSC: hematopoietic stem cells
HTS: high throughput screening
IC_50_: half maximal inhibitory concentration
iHLCs: induced hepatocyte like cells
iPSCs: induced pluripotent stem cells
MMV: Medicines for Malaria Venture
NAD: nicotinamide adenine dinucleotide
PB-PK: physiologically based pharmacokinetic
PK-PD: pharmacokinetic/pharmacodynamic
pLDH: parasite lactate dehydrogenase
PRISMA: preferred reporting items for systematic reviews and meta-analyses
SRB1: scavenger receptor class B type I
Tris-HCL/SDS: tris hydrochloride/sodium dodecyl sulfate
WHO: World Health Organization

## References

[B1] Adjalley S. H. Johnston G. L. Li T. Eastman R. T. Ekland E. H. Eappen A. G. (2011). Quantitative, assessment of *Plasmodium falciparum* sexual development reveals potent transmission-blocking activity by methylene blue. *Proc. Natl. Acad. Sci. U.S.A.* 108 E1214–E1223. 10.1073/pnas.1112037108 22042867 PMC3223476

[B2] Akala H. M. Eyase F. L. Cheruiyot A. C. Omondi A. A. Ogutu B. R. Waters N. C. (2011). Antimalarial drug sensitivity profile of western Kenya *Plasmodium falciparum* field isolates determined by a SYBR Green I in vitro assay and molecular analysis. *Am. J. Trop. Med. Hyg.* 85 34–41. 10.4269/ajtmh.2011.10-0674 21734121 PMC3122340

[B3] Bacon D. J. Jambou R. Fandeur T. Le Bras J. Wongsrichanalai C. Fukuda M. M. (2007). World antimalarial resistance network (WARN) II: in vitro antimalarial drug susceptibility. *Malar. J.* 6:120. 10.1186/1475-2875-6-120 17822533 PMC2008206

[B4] Baelmans R. Deharo E. Muñoz V. Sauvain M. Ginsburg H. (2000). Experimental conditions for testing the inhibitory activity of chloroquine on the formation of beta-hematin. *Exp. Parasitol.* 96 243–248. 10.1006/expr.2000.4558 11162377

[B5] Baker D. A. (2010). Malaria gametocytogenesis. *Mol. Biochem. Parasitol.* 172 57–65. 10.1016/j.molbiopara.2010.03.019 20381542 PMC2880792

[B6] Baniecki M. L. Wirth D. F. Clardy J. (2007). High-throughput *Plasmodium falciparum* growth assay for malaria drug discovery. *Antimicrob. Agents Chemother.* 51 716–723. 10.1128/AAC.01144-06 17116676 PMC1797774

[B7] Basco L. K. (2007). *Field Application of in vitro Assays for the Sensitivity of Human Malaria Parasites to Antimalarial Drugs.* Geneva: World Health Organization.

[B8] Basco L. K. Marquet F. Makler M. M. Le Bras J. (1995). *Plasmodium falciparum* and *Plasmodium vivax*: lactate dehydrogenase activity and its application for drug susceptibility assay. *Exp. Parasitol.* 80 260–271. 10.1006/expr.1995.1032 7895836

[B9] Basilico N. Pagani E. Monti D. Olliaro P. Taramelli D. (1998). A microtitre-based method for measuring the haem polymerization inhibitory activity (HPIA) of antimalarial drugs. *J. Antimicrob. Chemother.* 42 55–60. 10.1093/jac/42.1.55 9700528

[B10] Bennett T. N. Paguio M. Gligorijevic B. Seudieu C. Kosar A. D. Davidson E. (2004). Novel, rapid, and inexpensive cell-based quantification of antimalarial drug efficacy. *Antimicrob. Agents Chemother.* 48 1807–1810. 10.1128/AAC.48.5.1807-1810.2004 15105139 PMC400551

[B11] Berger B. J. Bendrat K. Cerami A. (1995). High-performance liquid chromatographic analysis of biological and chemical heme polymerization. *Anal. Biochem.* 231 151–156. 10.1006/abio.1995.1514 8678294

[B12] Bergmann-Leitner E. S. Mease R. M. Duncan E. H. Khan F. Waitumbi J. Angov E. (2008). Evaluation of immunoglobulin purification methods and their impact on quality and yield of antigen-specific antibodies. *Mal. J.* 7:129. 10.1186/1475-2875-7-129 18625058 PMC2490700

[B13] Borlon C. Russell B. Sriprawat K. Suwanarusk R. Erhart A. Renia L. (2012). Cryopreserved *Plasmodium vivax* and cord blood reticulocytes can be used for invasion and short term culture. *Int. J. Parasitol.* 42 155–160. 10.1016/j.ijpara.2011.10.011 22240310 PMC3438882

[B14] Bousema T. Okell L. Shekalaghe S. Griffin J. T. Omar S. Sawa P. (2010). Revisiting the circulation time of *Plasmodium falciparum* gametocytes: molecular detection methods to estimate the duration of gametocyte carriage and the effect of gametocytocidal drugs. *Malar. J.* 9:136. 10.1186/1475-2875-9-136 20497536 PMC2881938

[B15] Brasseur P. Agnamey P. Moreno A. Druilhe P. (2001). Evaluation of in vitro drug sensitivity of antimalarials for *Plasmodium falciparum* using a colorimetric assay (DELI-microtest). *Med. Trop. (Mars)* 61 545–547. 11980406

[B16] Brown W. M. Yowell C. A. Hoard A. Vander Jagt T. A. Hunsaker L. A. Deck L. M. (2004). Comparative structural analysis and kinetic properties of lactate dehydrogenases from the four species of human malarial parasites. *Biochemistry* 43 6219–6229. 10.1021/bi049892w 15147206

[B17] Buchholz K. Burke T. A. Williamson K. C. Wiegand R. C. Wirth D. F. Marti M. (2011). A high-throughput screen targeting malaria transmission stages opens new avenues for drug development. *J. Infect. Dis.* 203 1445–1453. 10.1093/infdis/jir037 21502082 PMC3080890

[B18] Chavalitshewinkoon-Petmitr P. Pongvilairat G. Auparakkitanon S. Wilairat P. (2000). Gametocytocidal activity of pyronaridine and DNA topoisomerase II inhibitors against multidrug-resistant *Plasmodium falciparum* in vitro. *Parasitol. Int.* 48 275–280. 10.1016/S1383-5769(99)00028-8 10725690

[B19] Chulay J. D. Haynes J. D. Diggs C. L. (1983). *Plasmodium falciparum*: assessment of in vitro growth by [3H] hypoxanthine incorporation. *Exp. Parasitol.* 55 138–146. 10.1016/0014-4894(83)90007-3 6337059

[B20] Chutmongkonkul M. Maier W. A. Seitz H. M. (1992). A new model for testing gametocytocidal effects of some antimalarial drugs on *Plasmodium falciparum* in vitro. *Ann. Trop. Med. Parasitol.* 86 207–215. 10.1080/00034983.1992.11812656 1449270

[B21] Crabb B. S. (2002). Transfection technology and the study of drug resistance in the malaria parasite *Plasmodium falciparum*. *Drug Resist. Updat.* 5 126–130. 10.1016/S1368-7646(02)00085-7 12237080

[B22] Cui L. Miao J. Cui L. (2007). Cytotoxic effect of curcumin on malaria parasite *Plasmodium falciparum*: inhibition of histone acetylation and generation of reactive oxygen species. *Antimicrob. Agents Chemother.* 51 488–494. 10.1128/AAC.01238-06 17145789 PMC1797756

[B23] Cui L. Miao J. Wang J. Li Q. Cui L. (2008). *Plasmodium falciparum*: development of a transgenic line for screening antimalarials using firefly luciferase as the reporter. *Exp. Parasitol.* 120 80–87. 10.1016/j.exppara.2008.05.003 18579134 PMC2559859

[B24] D’Alessandro S. Silvestrini F. Dechering K. Corbett Y. Parapini S. Timmerman M. (2013). A *Plasmodium falciparum* screening assay for anti-gametocyte drugs based on parasite lactate dehydrogenase detection. *J. Antimicrob. Chemother.* 68 2048–2058. 10.1093/jac/dkt165 23645588

[B25] De Clercq E. (2005). Recent highlights in the development of new antiviral drugs. *Curr. Opin. Microbiol.* 8 552–560. 10.1016/j.mib.2005.08.010 16125443 PMC7108330

[B26] Deharo E. García R. N. Oporto P. Gimenez A. Sauvain M. Jullian V. (2002). A non-radiolabelled ferriprotoporphyrin IX biomineralisation inhibition test for the high throughput screening of antimalarial compounds. *Exp. Parasitol.* 100 252–256. 10.1016/S0014-4894(02)00027-9 12128052

[B27] Dembélé L. Franetich J. F. Lorthiois A. Gego A. Zeeman A. M. Kocken C. H. (2014). Persistence and activation of malaria hypnozoites in long-term primary hepatocyte cultures. *Nat. Med.* 20 307–312. 10.1038/nm.3461 24509527

[B28] Desakorn V. Silamut K. Angus B. Sahassananda D. Chotivanich K. Suntharasamai P. (1997). Semi-quantitative measurement of *Plasmodium falciparum* antigen PfHRP2 in blood and plasma. *Trans. R. Soc. Trop. Med. Hyg.* 91 479–483. 10.1016/S0035-9203(97)90292-3 9373661

[B29] Desjardins R. E. Canfield C. J. Haynes J. D. Chulay J. D. (1979). Quantitative assessment of antimalarial activity in vitro by a semiautomated microdilution technique. *Antimicrob. Agents Chemother.* 16 710–718. 10.1128/AAC.16.6.710 394674 PMC352941

[B30] Dondorp A. M. Nosten F. Yi P. Das D. Phyo A. P. Tarning J. (2009). Artemisinin resistance in *Plasmodium falciparum* malaria. *N. Engl. J. Med.* 361 455–467. 10.1056/NEJMoa0808859 19641202 PMC3495232

[B31] Druilhe P. Moreno A. Blanc C. Brasseur P. H. Jacquier P. (2001). A colorimetric *in vitro* drug sensitivity assay for *Plasmodium falciparum* based on a highly sensitive double-site lactate dehydrogenase antigen-capture enzyme-linked immunosorbent assay. *Am. J. Trop. Med. Hyg.* 64 233–241. 11463109 10.4269/ajtmh.2001.64.233

[B32] Elabbadi N. Ancelin M. L. Vial H. J. (1992). Use of radioactive ethanolamine incorporation into phospholipids to assess in vitro antimalarial activity by the semiautomated microdilution technique. *Antimicrob. Agents Chemother.* 36 50–55. 10.1128/AAC.36.1.50 1590699 PMC189224

[B33] Fernandez-Becerra C. Lelievre J. Ferrer M. Anton N. Thomson R. Peligero C. (2013). Red blood cells derived from peripheral blood and bone marrow CD34? human haematopoietic stem cells are permissive to *Plasmodium* parasites infection. *Mem. Inst. Oswaldo Cruz* 108 801–803. 10.1590/0074-0276108062013019 24037205 PMC3970681

[B34] Fidock D. A. Rosenthal P. J. Croft S. L. Brun R. Nwaka S. (2004). Antimalarial drug discovery: efficacy models for compound screening. *Nat. Rev. Drug Discov.* 3 509–520. 10.1038/nrd1416 15173840

[B35] Fleck S. L. Pudney M. Sinden R. E. (1996). The effect of atovaquone (566C80) on the maturation and viability of *Plasmodium falciparum* gametocytes in vitro. *Trans. R. Soc. Trop. Med. Hyg.* 90 309–312. 10.1016/S0035-9203(96)90266-7 8758088

[B36] Francis S. E. Sullivan D. J. Jr. Goldberg D. E. (1997). Hemoglobin metabolism in the malaria parasite *Plasmodium falciparum*. *Annu. Rev. Microbiol.* 51 97–123. 10.1146/annurev.micro.51.1.97 9343345

[B37] Geary T. G. Divo A. A. Jensen J. B. (1983). An in vitro assay system for the identification of potential antimalarial drugs. *J. Parasitol.* 69 577–583. 10.2307/3281373 6355423

[B38] Gego A. Silvie O. Franetich J. F. Farhati K. Hannoun L. Luty A. J. (2006). New approach for high-throughput screening of drug activity on *Plasmodium* liver stages. *Antimicrob. Agents Chemother.* 50 1586–1590. 10.1128/AAC.50.4.1586-1589.2006 16569892 PMC1426939

[B39] Gelb M. H. (2007). Drug discovery for malaria: a very challenging and timely endeavor. *Curr. Opin. Chem. Biol.* 11 440–445. 10.1016/j.cbpa.2007.05.038 17761335 PMC1993815

[B40] Giarratana M. C. Rouard H. Dumont A. Kiger L. Safeukui I. Le Pennec P. Y. (2011). Proof of principle for transfusion of in vitro-generated red blood cells. *Blood* 118 5071–5079. 10.1182/blood-2011-06-362038 21885599 PMC3217398

[B41] Golenda C. F. Li J. Rosenberg R. (1997). Continuous in vitro propagation of the malaria parasite *Plasmodium vivax*. *Proc. Natl. Acad. Sci. U.S.A.* 94 6786–6791. 10.1073/pnas.94.13.6786 9192643 PMC21236

[B42] Gómez-Lechón M. J. Donato M. T. Castell J. V. Jover R. (2003). Human hepatocytes as a tool for studying toxicity and drug metabolism. *Curr. Drug Metab.* 4 292–312. 10.2174/1389200033489424 12871046

[B43] Grimberg B. T. Scheetz E. A. Erickson J. J. Bales J. M. David M. Daum-Woods K. (2012). Increased reticulocyte count from cord blood samples using hypotonic lysis. *Exp. Parasitol.* 132 304–307. 10.1016/j.exppara.2012.07.006 22841523

[B44] Hernandez L. Kodali S. Cully D. Singh S. Wang J. (2006). A target-specific whole cell assay for antibacterial drug discovery. *Protoc. Exch.* 10.1038/nprot.2006.130

[B45] Hobbs C. Duffy P. (2011). Drugs for malaria: something old, something new, something borrowed. *F1000 Biol. Rep.* 3:24. 10.3410/B3-24 22076126 PMC3206709

[B46] Howard R. J. Uni S. Aikawa M. Aley S. B. Leech J. H. Lew A. M. (1986). Secretion of a malarial histidine-rich protein (PfHRPII) from *Plasmodium falciparum*-infected erythrocytes. *J. Cell Biol.* 103 1269–1277. 10.1083/jcb.103.4.1269 3533951 PMC2114335

[B47] Hughes J. P. Rees S. Kalindjian S. B. Philpott K. L. (2011). Principles of early drug discovery. *Br. J. Pharmacol.* 162 1239–1249. 10.1111/j.1476-5381.2010.01127.x 21091654 PMC3058157

[B48] Hyde J. E. (2007). Drug-resistant malaria - an insight. *FEBS J.* 274 4688–4698. 10.1111/j.1742-4658.2007.05999.x 17824955 PMC2720519

[B49] Kaiser A. Khomutov A. Simonian A. Agostinelli E. A. (2012). A rapid and robust assay for the determination of the amino acid hypusine as a possible biomarker for a high-throughput screening of antimalarials and for the diagnosis and therapy of different diseases. *Amino Acids* 42 1651–1659. 10.1007/s0072601108595 21360085

[B50] Khan T. van Brummelen A. C. Parkinson C. J. Hoppe H. C. (2012). ATP and luciferase assays to determine the rate of drug action in *in vitro* cultures of *Plasmodium falciparum*. *Malar. J.* 11 369. 10.1186/1475-2875-11-369 23134617 PMC3505462

[B51] Kosaisavee V. Suwanarusk R. Nosten F. Kyle D. E. Barrends M. Jones J. (2006). *Plasmodium vivax*: isotopic, PicoGreen, and microscopic assays for measuring chloroquine sensitivity in fresh and cryopreserved isolates. *Exp. Parasitol.* 114 34–39. 10.1016/j.exppara.2006.02.006 16545375

[B52] Kurosawa Y. Dorn A. Kitsuji-Shirane M. Shimada H. Satoh T. Matile H. (2000). Hematin polymerization assay as a high-throughput screen for identification of new antimalarial pharmacophores. *Antimicrob. Agents Chemother.* 44 2638–2644. 10.1128/AAC.44.10.2638-2644.2000 10991837 PMC90128

[B53] Lelièvre J. Almela M. J. Lozano S. Miguel C. Franco V. Leroy D. (2012). Activity of clinically relevant antimalarial drugs on *Plasmodium falciparum* mature gametocytes in an ATP bioluminescence “transmission blocking” assay. *PLOS ONE* 7:e35019. 10.1371/journal.pone.0035019 22514702 PMC3325938

[B54] Li A. P. Kedderis G. L. (1997). Primary hepatocyte culture as an experimental model for the evaluation of interactions between xenobiotics and drug-metabolizing enzymes. *Chem. Biol. Interact.* 107 1–3. 10.1016/S0009-2797(97)00069-0 9402945

[B55] Liberati A. Altman D. G. Tetzlaff J. Mulrow C. Gøtzsche P. C. Ioannidis J. P. (2009). The PRISMA statement for reporting systematic reviews and meta-analyses of studies that evaluate healthcare interventions: explanation and elaboration. *BMJ* 339:b2700. 10.1136/bmj.b2700 19622552 PMC2714672

[B56] Lucantoni L. Avery V. M. (2012). Whole-cell in vitro screening for gametocytocidal compounds. *Future Med. Chem.* 4 2337–2360. 10.4155/fmc.12.188 23234555

[B57] Lucantoni L. Duffy S. Adjalley S. H. Fidock D. A. Avery V. M. (2013). Identification of MMV malaria box inhibitors of *Plasmodium falciparum* early-stage gametocytes using a luciferase-based high-throughput assay. *Antimicrob. Agents Chemother.* 57 6050–6062. 10.1128/AAC.00870-13 24060871 PMC3837862

[B58] Lucantoni L. Fidock D. A. Avery V. M. (2016). Luciferase-based, high-throughput assay for screening and profiling transmission-blocking compounds against *Plasmodium falciparum* gametocytes. *Antimicrob. Agents Chemother.* 60 2097–2107. 10.1128/AAC.01949-15 26787698 PMC4808229

[B59] Lucantoni L. Loganathan S. Avery V. M. (2017). The need to compare: assessing the level of agreement of three high-throughput assays against *Plasmodium falciparum* mature gametocytes. *Sci. Rep.* 7:45992. 10.1038/srep45992 28378767 PMC5380998

[B60] Makler M. T. Hinrichs D. J. (1993). Measurement of the lactate dehydrogenase activity of *Plasmodium falciparum* as an assessment of parasitemia. *Am. J. Trop. Med. Hyg.* 48 205–210. 10.4269/ajtmh.1993.48.205 8447524

[B61] Makler M. T. Ries J. M. Williams J. A. Bancroft J. E. Piper R. C. Gibbins B. L. (1993). Parasite lactate dehydrogenase as an assay for drug sensitivity. *Am. J. Trop. Med. Hyg.* 48 739–741. 10.4269/ajtmh.1993.48.739 8333566

[B62] March S. Ng S. Velmurugan S. Galstian A. Shan J. Logan D. J. (2013). A microscale human liver platform that supports the hepatic stages of *Plasmodium falciparum* and *vivax*. *Cell Host Microbe* 14 104–115. 10.1016/j.chom.2013.06.005 23870318 PMC3780791

[B63] Martín-Jaular L. Elizalde-Torrent A. Thomson-Luque R. Ferrer M. Segovia J. C. Herreros-Aviles E. (2013). Reticulocyte-prone malaria parasites predominantly invade CD71hi immature cells: implications for the development of an in vitro culture for *Plasmodium vivax*. *Malar. J.* 12:434. 10.1186/1475-2875-12-434 24289105 PMC4220676

[B64] Mazier D. Landau I. Druilhe P. Miltgen F. Guguen-Guillouzo C. Baccam D. (1984). Cultivation of the liver forms of *Plasmodium vivax* in human hepatocytes. *Nature* 307 367–369. 10.1038/307367a0 6363939

[B65] Ndiaye D. Patel V. Demas A. LeRoux M. Ndir O. Mboup S. (2010). A non-radioactive DAPI-based high-throughput in vitro assay to assess *Plasmodium falciparum* responsiveness to antimalarials–increased sensitivity of *P. falciparum* to chloroquine in Senegal. *Am. J. Trop. Med. Hyg.* 82 228–230. 10.4269/ajtmh.2010.09-0470 20133997 PMC2813162

[B66] Ng S. Schwartz R. E. March S. Galstian A. Gural N. Shan J. (2015). Human iPSC-derived hepatocyte-like cells support *Plasmodium* liver-stage infection in vitro. *Stem Cell Rep.* 4 348–359. 10.1016/j.stemcr.2015.01.002 25660406 PMC4375936

[B67] Noedl H. Faiz M. A. Yunus E. B. Rahman M. R. Hossain M. A. Samad R. (2003a). Drug-resistant malaria in Bangladesh: an in vitro assessment. *Am. J. Trop. Med. Hyg.* 68 140–142. 12641401

[B68] Noedl H. Se Y. Schaecher K. Smith B. L. Socheat D. Fukuda M. M. (2008). Evidence of artemisinin-resistant malaria in western Cambodia. *N. Engl. J. Med.* 359 2619–2620. 10.1056/NEJMc0805011 19064625

[B69] Noedl H. Wernsdorfer W. H. Miller R. S. Wongsrichanalai C. (2002). Histidine rich protein II, a novel approach to antimalarial drug susceptibility testing. *Antimicrob. Agents Chemother.* 46 1658–1664. 10.1128/AAC.46.6.1658-1664.2002 12019072 PMC127202

[B70] Noedl H. Wongsrichanalai C. Wernsdorfer W. H. (2003b). Malaria drug-sensitivity testing: new assays, new perspectives. *Trends Parasitol.* 19 175–181. 12689648 10.1016/s1471-4922(03)00028-x

[B71] Nogueira F. (2010). Methods for assessment of antimalarial activity in the different phases of the *Plasmodium* life cycle. *Rev. Pan Amaz. Saude* 1 109–124. 10.5123/S2176-62232010000300015

[B72] Noulin F. (2016). Malaria modeling: in vitro stem cells vs in vivo models. *World J. Stem Cells* 8 88–100. 10.4252/wjsc.v8.i3.88 27022439 PMC4807312

[B73] Noulin F. Borlon C. van den Eede P. Boel L. Verfaillie C. M. D’Alessandro U. (2012). Cryopreserved reticulocytes derived from hematopoietic stem cells can be invaded by cryopreserved *Plasmodium vivax* isolates. *PLOS ONE* 7:e40798. 10.1371/journal.pone.0040798 22844411 PMC3402485

[B74] Pandey A. V. Tekwani B. L. Singh R. L. Chauhan V. S. (1999). Artemisinin, an endoperoxide antimalarial, disrupts the hemoglobin catabolism and heme detoxification systems in malarial parasite. *J. Biol. Chem.* 274 19383–19388. 10.1074/jbc.274.27.19383 10383451

[B75] Panichakul T. Sattabongkot J. Chotivanich K. Sirichaisinthop J. Cui L. Udomsangpetch R. (2007). Production of erythropoietic cells in vitro for continuous culture of *Plasmodium vivax*. *Int. J. Parasitol.* 37 1551–1557. 10.1016/j.ijpara.2007.05.009 17610880

[B76] Parapini S. Basilico N. Pasini E. Egan T. J. Olliaro P. Taramelli D. (2000). Standardization of the physicochemical parameters to assess in vitro the beta-hematin inhibitory activity of antimalarial drugs. *Exp. Parasitol.* 96 249–256. 10.1006/expr.2000.4583 11162378

[B77] Peatey C. L. Spicer T. P. Hodder P. S. Trenholme K. R. Gardiner D. L. (2011). A high-throughput assay for the identification of drugs against late-stage *Plasmodium falciparum* gametocytes. *Mol. Biochem. Parasitol.* 180 127–131. 10.1016/j.molbiopara.2011.09.002 21939693

[B78] Phyo A. P. Nkhoma S. Stepniewska K. Ashley E. A. Nair S. McGready R. (2012). Emergence of artemisinin-resistant malaria on the western border of Thailand: a longitudinal study. *Lancet* 379 1960–1966. 10.1016/S0140-6736(12)60484-X 22484134 PMC3525980

[B79] Quashie N. B. de Koning H. P. Ranford-Cartwright L. C. (2006). An improved and highly sensitive microfluorimetric method for assessing susceptibility of *Plasmodium falciparum* to antimalarial drugs *in vitro*. *Malar. J.* 5:95. 10.1186/1475-2875-5-95 17076900 PMC1657019

[B80] Quignot N. Bois F. Y. (2013). A computational model to predict rat ovarian steroid secretion from in vitro experiments with endocrine disruptors. *PLOS ONE* 8:e53891. 10.1371/journal.pone.0053891 23326527 PMC3543310

[B81] Rebelo M. Sousa C. Shapiro H. M. Mota M. M. Grobusch M. P. Hänscheid T. (2013). A novel flow cytometric hemozoin detection assay for real-time sensitivity testing of *Plasmodium falciparum*. *PLOS ONE* 8:e61606. 10.1371/journal.pone.0061606 23637865 PMC3634823

[B82] Rieckmann K. H. Campbell G. H. Sax L. J. Mrema J. E. (1978). Drug sensitivity of *Plasmodium falciparum*. An in-vitro microtechnique. *Lancet* 1 22–30. 10.1016/S0140-6736(78)90365-3 74500

[B83] Rieckmann K. H. McNamara J. V. Frischer H. Stockert T. A. Carson P. E. Powell R. D. (1968). Effects of chloroquine, quinine, and cycloguanil upon the maturation of asexual erythrocytic forms of two strains of *Plasmodium falciparum* in vitro. *Am. J. Trop. Med. Hyg.* 17 661–671. 10.4269/ajtmh.1968.17.661 4877713

[B84] Roncales M. Vidal-Mas J. Leroy D. Herreros E. (2012). Comparison and optimization of different methods for the in vitro production of *Plasmodium falciparum* gametocytes. *J. Parasitol. Res.* 2012:927148. 10.1155/2012/927148 22523643 PMC3317192

[B85] Rothman S. S. (2002). *Lessons from the Living Cell: The Culture of Science and the Limits of Reductionism.* New York, NY: McGraw-Hill.

[B86] Sanchez B. A. Varotti F. P. Rodrigues F. G. Carvalho L. H. (2007). Validation of a *Plasmodium falciparum* parasite transformed with green fluorescent protein for antimalarial drug screening. *J. Microbiol. Methods* 69 518–522. 10.1016/j.mimet.2007.03.001 17466399

[B87] Sherman I. W. (1961). Heterogeneity of lactic dehydrogenase in avian malaria (*Plasmodium lophurae*). *J. Exp. Med.* 114 1049–1062. 10.1084/jem.114.6.1049 13911722 PMC2180406

[B88] Sherman I. W. (1977). Transport of amino acids and nucleic acid precursors in malarial parasites. *Bull. World Health Organ.* 55 211–225. 338180 PMC2366738

[B89] Sherman I. W. (1998). “Carbohydrate metabolism in asexual stages,” in *Malaria: Parasite Biology, Pathogenesis, and Protection* ed. Sherman I. W. (Washington, DC: ASM Press) 135–144.

[B90] Sinha S. Medhi B. Sehgal R. (2014). Challenges of drug-resistant malaria. *Parasite* 21 61. 10.1051/parasite/2014059 25402734 PMC4234044

[B91] Si-Tayeb K. Noto F. K. Nagaoka M. Li J. Battle M. A. Duris C. (2010). Highly efficient generation of human hepatocyte-like cells from induced pluripotent stem cells. *Hepatology* 51 297–305. 10.1002/hep.23354 19998274 PMC2946078

[B92] Slater A. F. Swiggard W. J. Orton B. R. Flitter W. D. Goldberg D. E. Cerami A. (1991). An iron-carboxylate bond links the heme units of malaria pigment. *Proc. Natl. Acad. Sci. U.S.A.* 88 325–329. 10.1073/pnas.88.2.325 1988933 PMC50803

[B93] Smalley M. E. (1977). *Plasmodium falciparum* gametocytes: the effect of chloroquine on their development. *Trans. R. Soc. Trop. Med. Hyg.* 71 526–529. 10.1016/0035-9203(77)90149-3 343314

[B94] Smilkstein M. Sriwilaijaroen N. Kelly J. X. Wilairat P. Riscoe M. (2004). Simple and inexpensive fluorescence-based technique for high-throughput antimalarial drug screening. *Antimicrob. Agents Chemother.* 48 1803–1806. 10.1128/AAC.48.5.1803-1806.2004 15105138 PMC400546

[B95] Sriwilaijaroen N. Kelly J. X. Riscoe M. Wilairat P. (2004). Cyquant cell proliferation assay as a fluorescence-based method for in vitro screening of antimalarial activity. *Southeast Asian J. Trop. Med. Public Health* 35 840–844. 15916078

[B96] Stojiljkovic I. Evavold B. D. Kumar V. (2001). Antimicrobial properties of porphyrins. *Expert Opin. Investig. Drugs* 10 309–320. 10.1517/13543784.10.2.309 11178343

[B97] Stone W. J. Churcher T. S. Graumans W. van Gemert G. J. Vos M. W. Lanke K. H. (2014). A scalable assessment of *Plasmodium falciparum* transmission in the standard membrane-feeding assay, using transgenic parasites expressing green fluorescent protein-luciferase. *J. Infect. Dis.* 210 1456–1463. 10.1093/infdis/jiu271 24829466

[B98] Sullivan A. D. Meshnick S. R. (1996). Haemozoin: identification and quantification. *Trends Parasitol.* 12 161–163. 10.1016/0169-4758(96)40001-1 15275227

[B99] Sung J. H. Esch M. B. Shuler M. L. (2010). Integration of in silico and in vitro platforms for pharmacokinetic-pharmacodynamic modeling. *Expert Opin. Drug Metab. Toxicol.* 6 1063–1081. 10.1517/17425255.2010.496251 20540627

[B100] Tanaka T. Q. Dehdashti S. J. Nguyen D. T. McKew J. C. Zheng W. Williamson K. C. (2013). A quantitative high throughput assay for identifying gametocytocidal compounds. *Mol. Biochem. Parasitol.* 188 20–25. 10.1016/j.molbiopara.2013.02.005 23454872 PMC3640759

[B101] Tanaka T. Q. Williamson K. C. (2011). A malaria gametocytocidal assay using oxidoreduction indicator, alamarBlue. *Mol. Biochem. Parasitol.* 177 160–163. 10.1016/j.molbiopara.2011.02.005 21316401 PMC3075389

[B102] Tekwani B. L. Walker L. A. (2005). Targeting the hemozoin synthesis pathway for new antimalarial drug discovery: technologies for in vitro beta-hematin formation assay. *Comb. Chem. High Throughput Screen.* 8 63–79. 10.2174/1386207053328101 15720198

[B103] Trager W. Jensen J. B. (1976). Human malaria parasites in continuous culture. *Science* 193 673–675. 10.1126/science.781840 781840

[B104] Tripathi A. K. Gupta A. Garg S. K. Tekwani B. L. (2001). In vitro beta-hematin formation assays with plasma of mice infected with *Plasmodium yoelii* and other parasite preparations: comparative inhibition with quinoline and endoperoxide antimalarials. *Life Sci.* 69 2725–2733. 10.1016/S0024-3205(01)01349-2 11720077

[B105] Tripathi A. K. Khan S. I. Walker L. A. Tekwani B. L. (2004). Spectrophotometric determination of de novo hemozoin/beta-hematin formation in an in vitro assay. *Anal. Biochem.* 325 85–91. 10.1016/j.ab.2003.10.016 14715288

[B106] van der Kolk M. De Vlas S. J. Saul A. van de Vegte-Bolmer M. Eling W. M. Sauerwein R. W. (2005). Evaluation of the standard membrane feeding assay (SMFA) for the determination of malaria transmission-reducing activity using empirical data. *Parasitology* 130 13–22. 10.1017/S0031182004006067 15700753

[B107] van Vianen P. H. Thaithong S. Reinders P. P. van Engen A. van der Keur M. Tanke H. J. (1990). Automated flow cytometric analysis of drug susceptibility of malaria parasites. *Am. J. Trop. Med. Hyg.* 43 602–607. 10.4269/ajtmh.1990.43.602 2267963

[B108] van Vianen P. H. van Engen A. Thaithong S. van der Keur M. Tanke H. J. van der Kaay H. J. (1993). Flow cytometric screening of blood samples for malaria parasites. *Cytometry* 14 276–280. 10.1002/cyto.990140307 7682494

[B109] Waki S. Tamura J. Jingu M. Adachi M. Suzuki M. (1986). A new technique for drug susceptibility tests for *Plasmodium falciparum* by ethidium bromide fluoroassay. *Trans. R. Soc. Trop. Med. Hyg.* 80 47–49. 10.1016/0035-9203(86)90192-6 3523864

[B110] Wein S. Maynadier M. Van Ba C. T. Cerdan R. Peyrottes S. Fraisse L. (2010). Reliability of antimalarial sensitivity tests depends on drug mechanisms of action. *J. Clin. Microbiol.* 48 1651–1660. 10.1128/JCM.02250-09 20220159 PMC2863886

[B111] Weisman J. L. Liou A. P. Shelat A. A. Cohen F. E. Guy R. K. DeRisi J. L. (2006). Searching for new antimalarial therapeutics amongst known drugs. *Chem. Biol. Drug Des.* 67 409–416. 10.1111/j.1747-0285.2006.00391.x 16882315 PMC1592519

[B112] Wernsdorfer W. H. Kouznetsov R. L. (1980). Drug-resistant malaria-occurrence, control, and surveillance. *Bull. World Health Organ.* 58 341–352. 6998589 PMC2395921

[B113] Wernsdorfer W. H. Payne D. (1988). “Drug sensitivity tests in malaria parasites,” in *Malaria: Principles and Practice of Malariology* eds Wernsdorfer W. H. McGregor I. A. (Edinburgh: Churchill Livingstone) 1765–1800.

[B114] WHO (2016). *World Malaria Report 2016.* Available at: http://www.who.int/malaria/publications/world-malaria-report-2016/report/en/

[B115] Yoon M. Campbell J. L. Andersen M. E. Clewell H. J. (2012). Quantitative in vitro to in vivo extrapolation of cell-based toxicity assay results. *Crit. Rev. Toxicol.* 42 633–652. 10.3109/10408444.2012.692115 22667820

